# “Taller and Shorter”: Human 3-D Spatial Memory Distorts Familiar Multilevel Buildings

**DOI:** 10.1371/journal.pone.0141257

**Published:** 2015-10-28

**Authors:** Thomas Brandt, Markus Huber, Hannah Schramm, Günter Kugler, Marianne Dieterich, Stefan Glasauer

**Affiliations:** 1 Clinical Neuroscience, Ludwig-Maximilians-University Munich, Germany; 2 German Center for Vertigo and Balance Disorders—IFBLMU (DSGZ), Ludwig-Maximilians-University Munich, Germany; 3 Department of Neurology, Ludwig-Maximilians-University Munich, Germany; 4 Munich Cluster for Systems Neurology (SyNergy), Munich, Germany; 5 Center for Sensorimotor Research; Ludwig-Maximilians-University Munich, Germany; 6 Bernstein Center for Computational Neuroscience; Ludwig-Maximilians-University Munich, Germany; 7 Hertie Foundation, Frankfurt a.M., Germany; University of Queensland, AUSTRALIA

## Abstract

Animal experiments report contradictory findings on the presence of a behavioural and neuronal anisotropy exhibited in vertical and horizontal capabilities of spatial orientation and navigation. We performed a pointing experiment in humans on the imagined 3-D direction of the location of various invisible goals that were distributed horizontally and vertically in a familiar multilevel hospital building. The 21 participants were employees who had worked for years in this building. The hypothesis was that comparison of the experimentally determined directions and the true directions would reveal systematic inaccuracy or dimensional anisotropy of the localizations. The study provides first evidence that the internal representation of a familiar multilevel building was distorted compared to the dimensions of the true building: vertically 215% taller and horizontally 51% shorter. This was not only demonstrated in the mathematical reconstruction of the mental model based on the analysis of the pointing experiments but also by the participants’ drawings of the front view and the ground plan of the building. Thus, in the mental model both planes were altered in different directions: compressed for the horizontal floor plane and stretched for the vertical column plane. This could be related to human anisotropic behavioural performance of horizontal and vertical navigation in such buildings.

## Introduction

Most swimming and flying species move and navigate in both the horizontal and vertical directions within their 3-D environment. They use path integration, continuously integrating direction and distance to envisaged goals. The honeybee integrates image motion across its eyes [1] Fish store horizontal and vertical components separately in their internal representation of space [2]. The vertical component may be less critical for survival in ground-based animals like rats or dogs compared to, e.g., bats [3]. However, animal experiments report contradictory findings as to whether there is a behavioral and neuronal anisotropy [4] exhibited in vertical and horizontal capabilities of spatial orientation and navigation, particularly with respect to the comparability of available reference frame cues [5]. Evidence was reported that rats give priority to the vertical dimension of space when relevant for orientation [6]. On the other hand, it has been shown that they exhibit a behavioural anisotropy when distributing their time freely between vertical and horizontal movements; they seem to prioritize the horizontal in both foraging and detour tasks [7]. The representation of the 3-D world is based on the activity of place cells in the hippocampus (information on position [8]), grid cells in the entorhinal cortex (information on distance [9,4]), and head direction cells (information on direction [10]). Rats climbing a wall showed impaired path integration by grid cells in the vertical domain [10]. The authors argue that these results can be simply accounted for by considering the different reference frames used by the rat. They present experimental evidence of the capability of rats to orient and navigate in the vertical domain [11].

**Fig 1:**
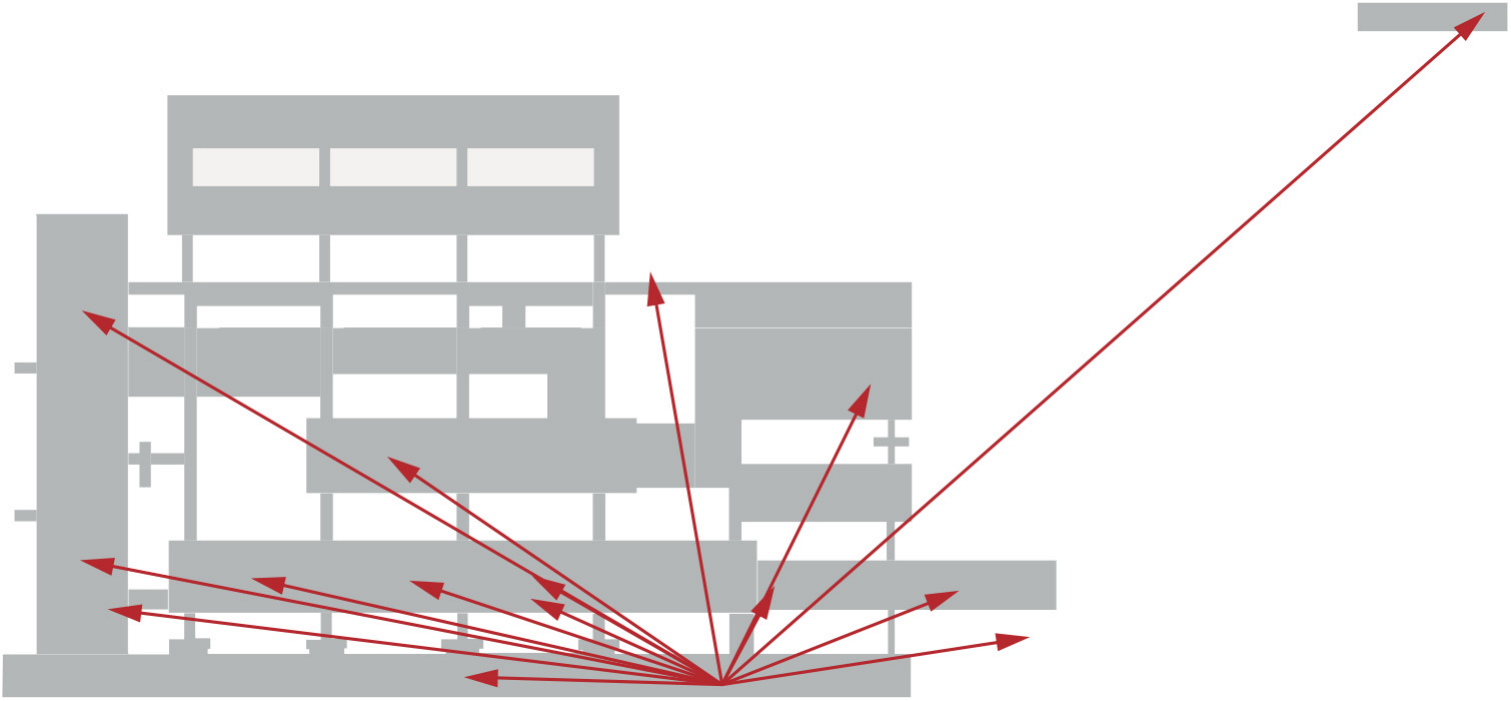
Pointing locations. Pointing locations as seen from above overlaid on an architectural ground plan of the hospital building. Note that the targets were not placed more toward the corners of the building but rather were distributed all over the building.

Our current study on 3-D orientation and spatial memory in humans was stimulated by an accidental observation made in dogs living for several days with their owners in a multilevel hotel [12]. The dogs had difficulties finding the right floor but correctly ran to the corresponding “right door” on the “wrong floor”. Our main question was whether humans also show an anisotropy of spatial memory for horizontal and vertical dimensions in a familiar multilevel building, i.e., a huge 15-floor university hospital (Fig 1). This can be investigated by two different experimental approaches: by a real navigation task in a familiar multilevel building [13] or by imagining and pointing to different landmarks within this building. Both experimental approaches have been shown to be complementary rather than equivalent and to yield different results in the horizontal plane of a virtual town [14]. Here we chose the second approach and designed a pointing experiment on the imagined 3-D direction of the location of various well-known but invisible goals that were distributed horizontally and vertically. The participants were employees who had worked for years in the building and were familiar with the localization of all targets. The hypothesis was that comparison of the assumed and the true directions might reveal systematic inaccuracy or dimensional anisotropy of localizations. Further, these localizations would allow the construction of a virtual 3-D model of our spatial memory and the internal representation of the building in our mind.

## Materials and Methods

All participants had worked regularly at the University Clinic for at least 5 years. They gave their verbal consent to participate in this study. They were just asked to participate in a direction-pointing experiment, which did not require written consent and required the participants to be naïve as to the aims and procedure of this psychophysical experiment. The consent was documented in the trial protocol. The experiment was approved by the Institutional Review Board of the ethics committee of the Ludwig-Maximilians University Munich in accordance with the Helsinki Declaration.

### Participants

Twenty-one healthy volunteers (8 females, age range 27–58, mean 44.3, SD 10.0) took part in this study(all were right-handed except for one female). All had normal or corrected-to-normal vision and no motor impairment that could affect finger-arm pointing. Participants had no neurological or psychiatric pathologies. They preferred to use the stairs to navigate between floors when less than five levels, but preferred the elevator for more than five levels.

### Design and procedure

All experiments were conducted in the same office where the enclosing walls prevented visibility of the targets (see Figs 1 and 3 for location). The examinee position for the pointing experiments was on the right side of the ground floor; it was chosen because this was a familiar meeting room. An optical tracking system (Qualisys, 8 cameras, frame rate 200 Hz) was used to track the pointing movements. Participants wore infrared reflective markers attached to their right index finger, right hand wrist and elbow, left and right shoulders, and forehead and faced along the short axis of the main building. The tracking system was installed to follow the whole movement range of all participants. All experiments were done under conditions of open eyes and a brightly illuminated test room.

For the calibration procedure the participants were instructed as follows: “In the first part of the experiment we will tell you at which corner of the room you have to look and point. Stand still, with lowered arms. Then point at the mentioned corner for 2 seconds, and then lower your arm.” The corners were defined as lower-right, upper-right, lower-middle, upper-middle, lower-left and upper left corner, corresponding to the heading position of the participants.

Pointing experiment: Instructions for the actual experiment were “In the second part, we will mention 15 various locations in the hospital such as wards, entrance, lecture halls in different horizontal directions and on different vertical levels (Table 1). The proportion of more vertically placed targets to targets placed more horizontally was balanced. Please point and look toward these targets. Imagine that a laser-pointer is attached to your index finger and its beam is to be directed to the assumed location of the mentioned target in the hospital. If you think you are pointing to the correct place, keep gaze and arm steady for 2 seconds and then lower your arm for the next condition.” The targets were distributed over the whole building (coordinate ranges along the long axis of main building -270 m to 226 m, along the short axis 2 m to 236 m, along the vertical axis -8 m to 38 m; absolute target distances 11 m to 358 m), and all participants were familiar with the locations (Fig 1).

**Table 1:** Names and coordinates of the target positions relative to the location of the participant.

Name	Abbr.	X [m]	Y [m]	Z [m]
Nurses’ room, Ward I2	I2	-36.85	-165.40	4.00
Nurses’ room, Ward G8	G8	-37.75	-66.43	24.50
Nurses’ room, Ward H8	H8	-36.00	-109.61	24.50
Conference room G12	G12	-29.66	-66.93	37.70
X-ray conference room	RK	-105.13	52.83	-4.00
Registration desk, Outpatients	NP	-79.69	-117.37	0.00
Lecture hall 1	HS1	-130.90	-225.12	0.00
Lecture hall 6	HS6	-43.20	-225.67	0.00
Main entrance	HE	-16.60	108.53	-4.00
Conference room 2, entrance	K2	-2.00	-90.36	0.00
Porter’s desk	PH	-236.08	269.62	-4.00
Hairdresser’s	FL	-7.98	7.05	0.00
Top end of escalator	RT	-32.22	84.19	0.00
Foto department, entrance	FA	-25.95	-215.94	-8.00
Emergency, ramp onset	NH	-144.75	-24.58	-8.00

Drawings: After the pointing experiment, participants were asked to draw a schematic front view and ground plan of the main building. The drawings were used to compare ratios of width, height, and length of the building with those derived from pointing (see sections below).

### Data processing

The recorded trajectories of the markers were labelled according to their attached joint offline in the QTM (Qualisys Tracking Manager) software. The end of each pointing episode was determined when the position of the wrist marker began to move downwards minus 0.2 s. The recorded and labelled data were exported to Matlab for further analysis. One hundred data points (0.5 s) before the end of a pointing episode were accumulated and averaged to calculate the pointing direction. Pointing direction was determined as follows (for methodological details see [15,16]). First, the mean error of the pointing direction toward the calibration corners was determined by using two different methods:

1. the direction starting at the mid-eye towards the pointing finger,
2. the direction defined by the shoulder towards the pointing finger.

The errors for the two methods were calculated for each participant and the method with the smallest error was used to define the participant’s individual pointing method. All calculations were done using Matlab Release 2013 or higher (Mathworks, USA) and its Optimization Toolbox.

### Calibration

The measured pointing directions to the corners of the room were used to calibrate the pointing angles of the experiment. Since the exact position of each corner relative to the head of the participant was known, it was possible to calculate the hypothetically exact pointing direction to the corners. The pointing directions are expressed in azimuth (*Az* horizontal) and elevation (*El* vertical) angles. The difference between an exact pointing direction and the measured pointing direction was used to calculate a calibration matrix *M*_*β*_, according to the ordinary least squares estimation:

$$M_{\beta }={\left(D^{T}\cdot D\right)}^{-1}\cdot D^{T}\cdot P_{c}$$ (1)

where *D* is a 3xN matrix containing the difference between exact and measured pointing angles (difference of *Az*, difference of *El*) and a vector of ones, which represents an offset. *P*_*c*_ is a matrix containing all measured pointing angles. The i-th column of *P*_*c*_ is a vector containing the pointing angles for the i-th target.

The calibration matrix allowed adjustment of the pointing angles for the experiment with:

$${\vec{p}}^{\prime }=M_{\beta }\cdot {\vec{p}}_{m}$$ (2)

where ${\vec{p}}_{m}$ is a vector containing the measured pointing angles during the experiment and ${\vec{p}}^{\prime }$ contains the calibrated pointing angles. The pointing angle was transformed into a unit vector with the appropriate pointing direction for further processing.

### Data analysis

Pointing angles indicated by azimuth and elevation were averaged over targets or subjects using circular statistics (CircStat toolbox for Matlab [17]).

To further understand the errors in pointing angles, a transformed building that best matched the subjects’ mean pointing directions was calculated. An optimization procudure (Matlab function lsqnonlin) was used to search for a linear transformation *T* of the normalized target directions ${\vec{t}}_{i}$ derived from the target coordinates that would minimize the angles between the normalized average pointing directions ${\vec{p}}_{i}$ and the transformed and normalized directions of the targets $\vec{t_{i}^{\prime }}=T\cdot {\vec{t}}_{i}$ so that

$$\sum_{i}\text{arccos}\left({\vec{p}}_{i}-T\cdot {\vec{t}}_{i}\right)=\text{min}$$ (3)

The linear transformation represented by the transformation matrix *T* allowed for rotation, shearing, and scaling. By adding a quadratic term, a second transformation function was generated to allow for a non-linear distortion. The same approach was used to calculate an individual transformation matrix for each participant corresponding to his or her respective pointing directions.

For 3-D modelling the ground plan of the building (Fig 1) was used to create a 3-D model with the AutoCAD software (Autodesk Inc., USA). The previously calculated general linear transformation was applied to the 3-D model using Matlab, and the aspect ratios of the resulting main building of the new model were determined by calculating its length, height, and width. To generate the figures, the transformed model was imported back to AutoCAD. The aspect ratios of length-height (L/H) and length-width (L/W) determined from the individual transformation matrices were then compared to the corresponding aspect ratios derived from the participants’ drawings.

## Results

According to the calibration procedure, approximately 57% of the participants (12 of 21) used the eye-finger pointing strategy. The average unsigned pointing error during the calibration procedure was 8.6±1.2°.

**Fig 2:**
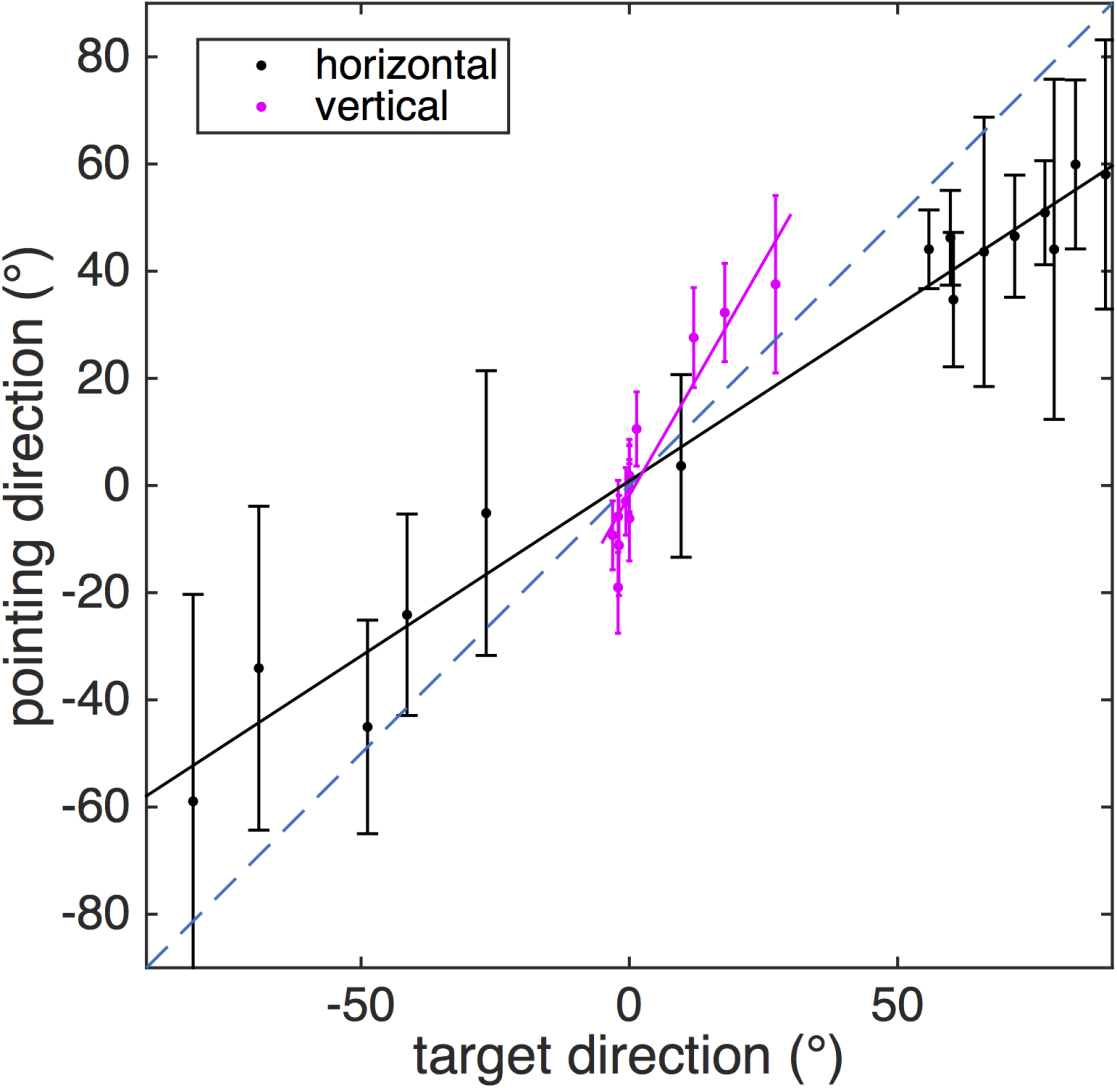
Pointing directions. Horizontal (azimuth, black dots) and vertical (elevation, red stars) mean pointing directions (error bar gives circular SD) plotted over mean target direction. Accurate pointing directions would agree with the dashed blue line. Orientation of the participants during the pointing task coincided with the short axis of the main building (tall red building part in Fig 3) and with zero azimuth angle. Note that horizontal pointing angles were on average smaller than target angles, indicating a reduction of perceived length of the building, while the opposite was the case for vertical angles, indicating an overestimation of the building’s height.

Averaged horizontal and vertical pointing directions over all subjects are shown in Fig 2 (see S1 File for individual data). Horizontal pointing directions were smaller than true target directions, indicating a compression of the perceived building along its long axis. In contrast, vertical pointing directions were larger, as if the height of the building had been overestimated. To quantify this relation the respective linear regressions were calculated for each subject. The average slope of horizontal pointing directions was 0.63±0.28 (including two outliers with negative slopes), for vertical pointing directions 1.73±0.48. Both average slopes are significantly different from unity (accurate pointing) and from each other (t-tests, all p<0.0001).

**Fig 3:**
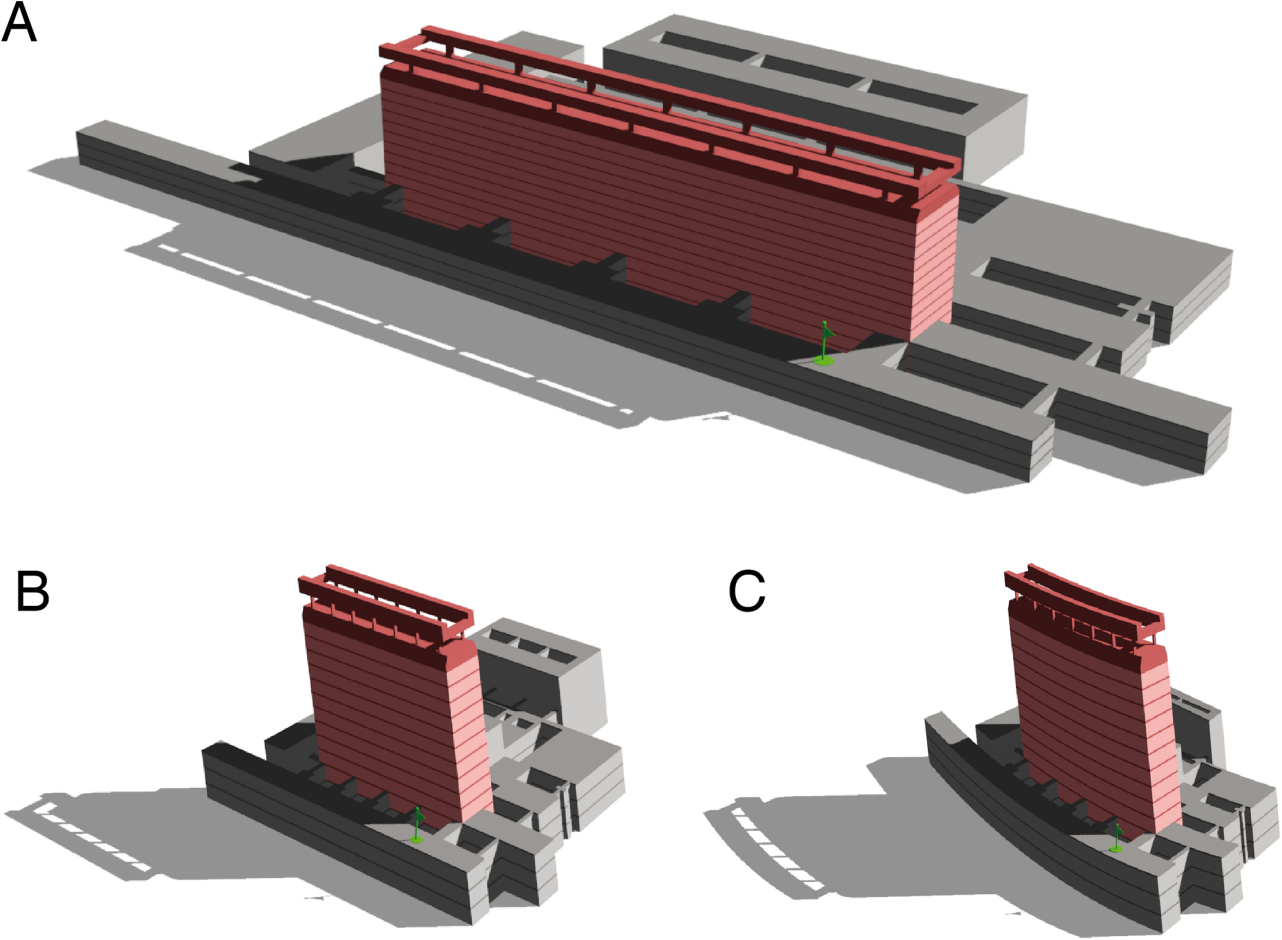
3-D model of the hospital building. A: original building. B: building reconstructed from the averaged pointing directions of the participants assuming a linear transformation of the building. C: building reconstructed from pointing but allowing for linear *and* quadratic transformation. Green flag indicates the position of the participants in the building. Red: main building. Note that the mental model appears to be compressed for the horizontal floor plane, stretched for the vertical column plane, and curved toward the subjective straight ahead.

**Fig 4:**
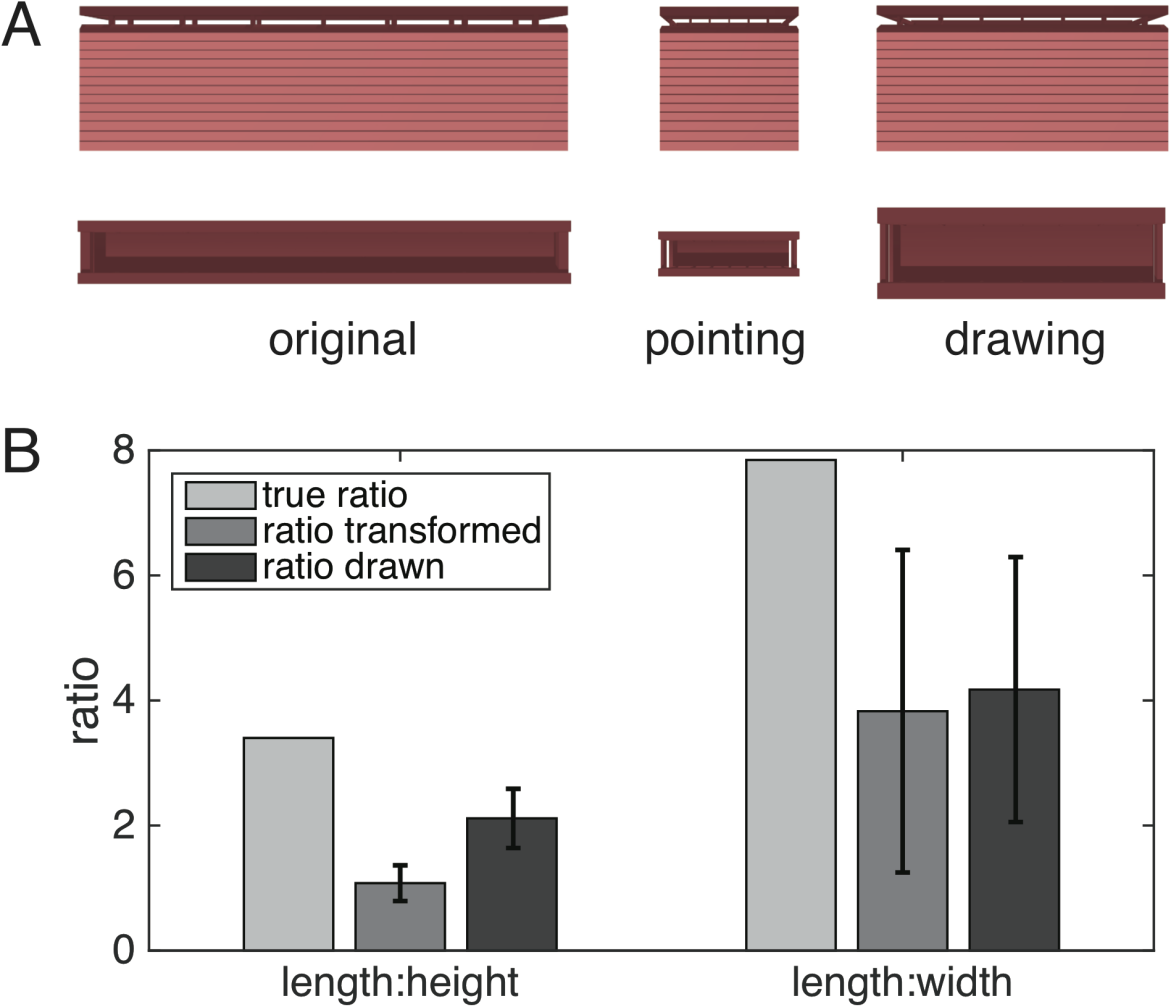
Aspect ratios of the main building. A: Front view (top row) and top view (bottom row) of the main building. Left column: true proportions of the building. Middle column: Transformed building according to averaged pointing directions. Right column: Transformed building according to averaged aspect ratios from participants’ drawings. Note that depictions are shown at equal height. B: Bar graph of aspect ratios. Left length:height, right: length:width. Left bar (light gray): true aspect ratio of the building, middle bar (gray): averaged ratios of transformed buildings according to individual pointing directions, right bar (dark gray): ratio taken from individual drawings. Error bars denote standard deviation.

From the pointing directions, it was estimated how the building would look, if pointing were accurate on average. To this end, a linear or linear-quadratic transformation was applied to the target coordinates, minimizing the difference between the transformed target directions and average pointing directions. The transformed buildings are shown in Fig 3 together with the original building for comparison. The linear transformation was then applied to the data of each participant in order to derive individual transformations. From the transformed buildings the aspect ratios of length-height and length-width were calculated and averaged. Fig 4 compares these values with the true aspect ratios and those derived from the drawings of the participants (see Table 2 for values). A repeated measures ANOVA of the ratios (factor 1: L/H or L/W; factor 2: from pointing or from drawing) shows a highly significant effect of L/H vs. L/W (F(1,20) = 47.7, p<0.0001) but no effect of drawing vs. pointing (F(1,20) = 3.17, p = 0.09 n.s.) or interaction between the two factors.

**Table 2:** Aspect ratios of the main building (see also Fig 3).

	length:height	length:width
True aspect ratio	3.40	7.85
Averaged individual pointing ratios	1.08±0.29	3.83±2.58
Ratio derived from averaged pointing data	1.03	3.19
Average individual drawing ratios	2.11±0.47	4.17±2.12

## Discussion

The major finding of this study was that the internal representation of the familiar multilevel building appeared to be distorted and convexly curved from the subject’s viewpoint: it seemed vertically taller and horizontally shorter (see Fig 3). This holds not only for the group average but for the great majority of single subjects tested (exceptions: one outlier in the pointing task and one in the drawing task, each for the ratio L/W). It was demonstrated both for the mathematical reconstruction based on the analysis of the pointing experiment and the drawings of the front view and ground plan of the building. Distortions of the internal model of the building were horizontally and vertically reciprocal, i.e., 215% taller and 51% shorter in the pointing experiment and 61% taller and 46% shorter in the drawings.

Our current understanding of neural representation of the surrounding 3-D space is based on the assumption that cognitive maps are continuously encoded by place cells, grid cells, and head direction cells [18]. The formation of such cognitive maps depends on a cascade of increasingly complex associative processes involving object location, subject location, and then the formation of building blocks for learning temporo-spatial sequences [19]. Orientation and navigation depend on the interplay of various cognitive processes [20]. One such process is landmark-based piloting, i.e., (1) identify individual landmarks, (2) use these landmarks to determine your (?) current position and heading, (3) access long-term knowledge about the spatial relationships between locations, and (4) use this knowledge to plan a route to the navigational goal [21]. The parahippocampal place area seems to be critical for landmark recognition, the retrosplenial/medial parietal region, for localization and orientation, and both medial temporal lobe and retrosplenial/medial parietal lobe regions, for long-term spatial knowledge [21].

Furthermore, the posterior hippocampus plays a significant role in both the encoding and retrieval of spatial memory, orientation, and navigation [22]. It has different functions than the anterior hippocampus; whereas the posterior hippocampus is mainly involved in cognitive functions, the anterior is more involved in emotional processes [23]. There is also a hippocampal separation for processing vestibular inputs (anterior hippocampus) and visual inputs (posterior hippocampus) [24]. The separation of vestibular and visual information in the hippocampal formation has a twofold functional consequence: missing input from one system may be partially substituted by the other, and the task-dependent sensorial weight can be shifted to the more reliable modality for navigation [24]. The latter is reflected by the structural and functional plasticity of the hippocampal formation observed in professional dancers and slackliners. They have smaller anterior (vestibular) volumes as a result of long-term suppression of destabilizing vestibular input, but larger posterior (visual) volumes as a result of increased utilization of visual cues for balance [25].

There seem to be several separate vestibular pathways connecting the hippocampus with the parietal cortex to subserve spatial learning and spatial memory [26]. The posterior parietal cortex and the parahippocampal place area are believed to integrate multisensory spatial representation and the planning of goal-directed movements [27,28,22]. It is not yet fully understood how the neural mechanisms in the parahippocampal place area, retrosplenial cortex, and medial temporal lobe interact to form a global representation of the 3-D world [21].

To the best of our knowledge, this study is the first to analyze the 3-D internal representation in humans of a complex building. This representation serves as a cognitive map not only for pointing but also for navigation. It remains open as to whether the participants formed a continuous vertical representation of the building’s floor plan, for we did not systematically interview them on this question. Their spontaneous reports differed considerably: some subjects said that they imagined the building’s shape from outside, others imagined parts or blocks of the building with the location of the particular invisible goal they had to point to, and a few worked in two steps, i.e., first imagining the location of the goal relative to the horizontal floor and then estimating the vertical position up or down, which was similar to the”direction strategy and floor strategy” [29]. We did not intend to contribute new knowledge about the cortical structures and functions involved in encoding spatial localizations. Instead, we were interested in the actual performance of retrieving landmarks in horizontal versus vertical dimensions in order to reconstruct an internal representation of the entire 3-D building. Earlier studies focused on how horizontal and vertical navigation influences spatial memory of virtual multi-floored environments [30] and of real buildings [31]. The learning effect of navigation in the horizontal (floor) plane on the spatial memory of a real building was better than was that along the vertical levels (column) The authors attributed this to the structural differences in the horizontal and vertical planes [31]. In virtual reality, however, horizontal (floor) recognition was not reliably superior to column recognition, but learning along a floor route produced a better spatial memory performance [30].

The resulting horizontally and vertically distorted internal representation is supported by the subjects’ drawings of the front view and ground plan of the building. The drawings, i.e., the imagination of the building, confirm the 3-D reconstruction: both planes are altered differently (compressed for the horizontal floor plane and stretched for the vertical column plane). While the compression observed in the horizontal plane can be related to general effects in magnitude estimation such as the regression effect [32], the vertical stretching shows quite the opposite tendency. Questions arise as to how to interpret these results with respect to the anisotropy of a weaker performance of navigation in the vertical versus the horizontal dimension, which was described in rodents [4,7], dogs [12], and humans [13]. PET measurements in the latter study showed increased glucose metabolism in the right hippocampus, bilateral retrosplenial cortex, and pontine tegmentum during horizontal navigation [13]. In contrast, vertical navigation activated the bilateral hippocampus and the vestibular insular cortex.

An anisotropic navigation performance may be due to the different cues provided during way finding on a horizontal floor versus the traversing of the building vertically by a stairwell. The stairwell provides fewer landmarks during locomotion and predominantly relies on path integration, i.e., the performed path is compared with the internal representation of the path along the floors. The horizontal plane provides many more landmarks on the way to the destination. These landmarks may allow us to re-compute the distance to the destination, which may correct for the shorter imagined distance of the initial route plan. This may be related to the specific strategies for navigating multilevel buildings: the direction strategy, which relies on routes that first head towards the horizontal position of the goal and a floor strategy that relies on routes that first head towards the vertical position of the goal [29]. These authors found that experienced subjects preferred the floor strategy, which also resulted in a better way-finding performance.

The imagined building in our study appeared to be convexly curved. This impression may be due to the eccentric position of the participant which causes a bias of the most distant targets inhorizontal directions toward the subjective straight ahead (see Fig 3).

The finding of our current study that an internal representation of a familiar multilevel building is distorted raises several questions. For example, does this representation depend on the subject’s position (viewpoint) and if so, how? Does the shape of the representation change during true locomotion or imagined locomotion within the building? This is relevant because of the so-called ‘Event-Horizon-Effect’. Previous research using virtual or real environments has revealed that walking through doorways causes a location-updating effect with a decline in memory, suggesting a segmentation of internal representations of space [33,34]. This is also reflected by grid cell behaviour in rat experiments in which the firing pattern was initially compartmentalized but after longer experience in the same environment reflected the connected environments [35].

## Supporting Information

S1 File Pointing data. Supplementary information contains a semicolon-delimited CSV data file with pointing data (in deg) generated by Matlab. Each row contains data of one participant (15 columns horizontal angles and 15 columns vertical angles, one column for each target, same order as in Table 1). Missing values are denoted by the string 'NaN'.
(XLS)
